# AP-1 elements in the promoter and second intron mediate endoplasmic reticulum stress-induced expression of the *GPAT3* gene

**DOI:** 10.1038/s41598-025-32503-y

**Published:** 2025-12-14

**Authors:** Daima Örd, Tiit Örd, Elina Gluboki, Tõnis Örd

**Affiliations:** 1 Institute of Genomics, University of Tartu, Tartu, Estonia; 2 Institute of Molecular and Cell Biology, University of Tartu, Tartu, Estonia

**Keywords:** ATF4, Transcriptional regulation, Lipid, Triglyceride accumulation, Integrated stress response, Cell biology, Molecular biology

## Abstract

An excessive accumulation of hepatic lipids is a characteristic feature of metabolic dysfunction-associated steatotic liver disease (MASLD) and its severe form, metabolic dysfunction-associated steatohepatitis (MASH). Acyl-CoA:glycerol-sn-3-phosphate acyltransferase 3 (GPAT3) and other members of the GPAT family are enzymes which play an important role in glycerolipid synthesis. Previous articles have reported that *GPAT3* mRNA and ER stress marker genes are upregulated in patients with MASH. Here, we study the regulatory mechanism of *GPAT3* gene expression in human hepatoma cells suffering from ER stress. Transcriptome profiling showed that among the genes implicated in the formation of glycerolipids, *GPAT3* is one of the most strongly activated genes in response to tunicamycin, an inducer of ER stress. CRISPR/Cas9-mediated disruption of *activating transcription factor 4* (*ATF4*) resulted in reduced *GPAT3* expression under ER stress. Luciferase reporter assays of *GPAT3* gene fragments encompassing ATF4 ChIP-seq peaks and mutational analysis revealed that activator protein-1 (AP-1) sites located in *GPAT3* promoter and intron 2 mediate the activation of transcription in response to ER stress and ATF4. CRISPR/Cas9-mediated deletion of the region containing AP-1 sites from *GPAT3* intron 2 caused a reduction of *GPAT3* expression and triglyceride content in both unstressed cells and under ER stress. Thus, the results indicate that the induction of *GPAT3* expression in response to ER stress is mediated by ATF4 via AP-1 elements in the promoter and second intron.

**Supplementary Information:**

The online version contains supplementary material available at 10.1038/s41598-025-32503-y.

## Introduction

The excess of free fatty acids, often associated with overnutrition, enhances lipogenesis and triglyceride synthesis within liver cells. Acyl-CoA:glycerol-sn-3-phosphate acyltransferases (GPATs) catalyze the first and rate-limiting step in the *de novo* synthesis of glycerophospholipids and triglycerides, the conversion of long-chain acyl-CoA and glycerol-3-phosphate to lysophosphatidic acid^1^. In mammals, four GPAT isoforms (GPAT1-4) have been identified. While GPAT1 and GPAT2 are mitochondrial, GPAT3 (previously known as AGPAT9) and GPAT4 are localized to the endoplasmic reticulum (ER), an organelle that carries out many crucial cell functions, such as protein synthesis, folding, modification and trafficking, lipid synthesis, and calcium storage^2–4^. The preservation of ER homeostasis is tightly controlled and homeostasis disruption triggers a set of intracellular signaling pathways known as the unfolded protein response (UPR), consisting of three branches: the PERK-eIF2α-ATF4 pathway of the integrated stress response (ISR), and the IRE1α-XBP1 and ATF6α pathways^5^. Chronic ER stress resulting from ER homeostasis disruption is central to the pathophysiology of a variety of human diseases, ranging from cancer and neurodegeneration to diabetes, obesity and metabolic dysfunction-associated steatotic liver disease (MASLD), formerly known as nonalcoholic fatty liver disease^6–8^.

*GPAT3* expression is activated by saturated fatty acids^9^, which in excess are toxic to cells and evoke cellular stress responses, including the UPR^10,11^. Analysis of liver biopsies has revealed that *GPAT3* mRNA^12^ as well as ER stress marker genes^13–15^ are upregulated in patients with metabolic dysfunction-associated steatohepatitis (MASH)^12^, the severe form of MASLD. MASLD and MASH are characterized by excessive accumulation of hepatic lipids, and MASH has the potential to progress to cirrhosis and hepatocellular carcinoma. Compared to subjects with metabolic syndrome but normal liver histology, MASLD and MASH cases were found to be associated with eIF2α phosphorylation, the hallmark of ISR activation^13^. Importantly, deletion of *GPAT3* in mice alleviates liver steatosis and insulin resistance in a model of severe lipodystrophy^16^ and attenuates corticosterone-induced fatty liver^17^. Recently, it was found that in hepatocellular carcinoma (HCC) cells, GPAT3 upregulation reprograms lipid metabolism, and contributes to acquired resistance to sorafenib, the standard treatment for advanced HCC^18^.

While it has been previously reported that ER stress alters lipid metabolism^19,20^, detailed knowledge about the regulation of *GPAT3* expression during ER stress is limited. In the current article, we characterize the mechanism of *GPAT3* expression regulation in HepG2 human hepatoma cells suffering from ER stress. We show that ATF4, the master regulator of gene expression of the ISR, activates *GPAT3* transcription in response to ER stress via activator protein-1 (AP-1) sites within the *GPAT3* promoter and the second intron. We further confirm that *GPAT3* gene expression is positively associated with the ER stress-dependent and -independent accumulation of triglycerides in HepG2 cells.

## Results

### *GPAT3* expression is upregulated in cells suffering from ER stress

**Fig. 1:**
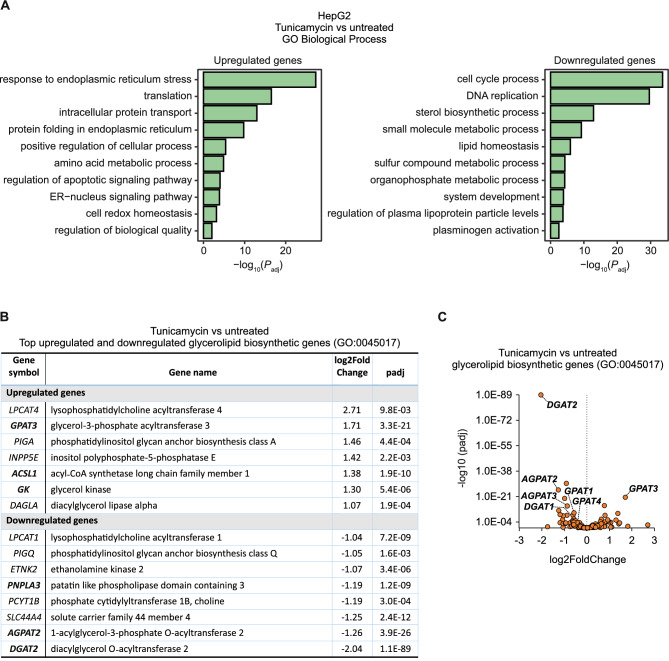
RNA-seq analysis of gene expression changes in HepG2 cells treated with tunicamycin. (**A**) Gene Ontology (GO) biological process enrichment analysis of differentially expressed genes in response to the exposure of the cells to tunicamycin. Top 500 most statistically significantly up- or downregulated genes were analyzed using gProfiler. The 10 most significant GO driver terms are shown for each direction. (**B**, **C**) Differential expression of genes belonging to the glycerolipid biosynthetic process (GO:0045017) between tunicamycin-treated HepG2 cells and untreated control. Glycerolipid pathway differentially expressed genes with absolute log_2_ fold change >1 and p_adj_ < 0.05 (p-value adjusted for multiple testing by the Benjamini-Hochberg procedure) (**B**), and scatterplot showing log_2_ fold change and -log_10_ p_adj_ of all glycerolipid pathway genes (**C**). In panel C, gene symbols in bold indicate the genes related to triglyceride metabolism. *Glycerol-3-phosphate acyltransferase 1* (*GPAT1*), *glycerol-3-phosphate acyltransferase 4* (*GPAT4*), *1-acylglycerol-3-phosphate O-acyltransferase 3* (*AGPAT3*), *diacylglycerol O-acyltransferase 1* (*DGAT1*).

**Fig. 2:**
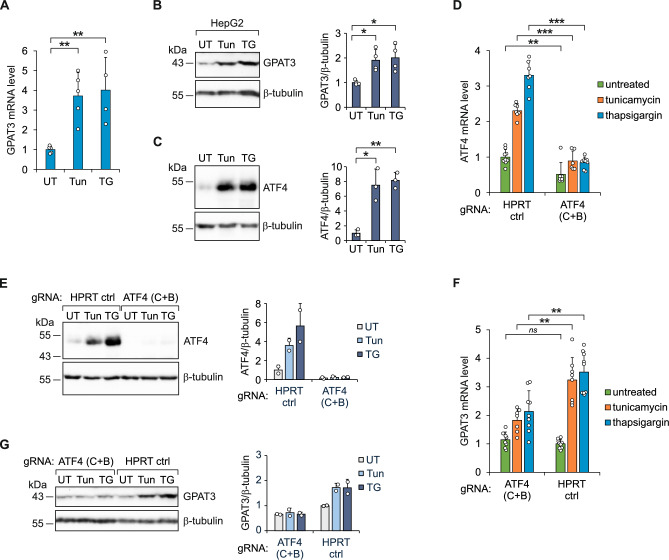
ATF4 mediates *GPAT3* induction in HepG2 cells in response to ER stress. (**A**, **B**) Quantification of *GPAT3* mRNA by RT-qPCR (**A**; *n* = 4-5) and detection of GPAT3 protein by immunoblot (**B**) in cells treated with tunicamycin (Tun) or thapsigargin (TG) for 17 h or left untreated (UT). In B, the representative immunoblot analysis (*n* = 4) and densitometry quantification of GPAT3 protein expression are shown. Data are presented as the means ± SD and expressed relative to the level of *GPAT3* mRNA (**A**) or protein (**B**) in untreated cells. (**C**) The representative immunoblot analysis (*n* = 3) and densitometry quantification of ATF4 protein level in cells treated with tunicamycin (Tun) or thapsigargin (TG) for 17 h or left untreated (UT). Data are presented as the means ± SD and expressed relative to the level of ATF4 protein in untreated cells. (**D**, **E**) The downregulation of *ATF4* expression by CRISPR-Cas9-based disruption of the *ATF4* gene. Quantification of *ATF4* mRNA by RT-qPCR (**D**; *n* = 6-9) and detection of ATF4 protein by immunoblot (**E**) in cells transfected with control gRNA (HPRT ctrl) or gRNAs designed to disrupt *ATF4* gene (ATF4 (C+B)) and treated with tunicamycin or thapsigargin for 17 h or left untreated. In E, the representative immunoblot analysis (*n* = 2) and densitometry quantification of ATF4 protein expression are shown. Data are presented as the means ± SD and expressed relative to the level of *ATF4* mRNA (**D**) or protein (**E**) in untreated cells transfected with control gRNA. (**F**, **G**) The downregulation of *GPAT3* expression by CRISPR-Cas9-based disruption of *ATF4* gene. Quantification of *GPAT3* mRNA by RT-qPCR (F; *n* = 7-9) and detection of GPAT3 protein by immunoblot (**G**) in cells transfected with gRNAs designed to disrupt *ATF4* gene (ATF4 (C+B)) or control gRNA (HPRT ctrl) and treated with tunicamycin or thapsigargin for 17 h or left untreated. In G, the representative immunoblot analysis (*n* = 2) and densitometry quantification of GPAT3 protein expression are shown. Data are presented as the means ± SD and expressed relative to the level of *GPAT3* mRNA (F) or protein (G) in untreated cells transfected with control gRNA. * *p* < 0.05, ** *p* < 0.005, *** *p* < 0.0005, *ns* (not significant) comparing the indicated conditions using two-tailed *t* tests followed by Holm-Bonferroni correction. Uncropped blots are presented in Supplementary Information 4.

In order to characterize the ER stress-induced changes in the expression of genes, HepG2 cells were exposed to tunicamycin, an inhibitor of N-linked glycosylation, and transcriptome profiling was carried out by RNA sequencing. The results showed that tunicamycin treatment significantly altered the expression level of 4317 genes (p_adj_ < 0.05), including 646 genes upregulated and 454 genes downregulated more than 2-fold (Supplementary Information 1 (Table S1)). Functional profiling of genes upregulated by tunicamycin revealed enrichment Gene Ontology (GO) biological processes such as the response to ER stress, intracellular protein transport and protein folding in the ER (Fig. 1A, Supplementary Information 2 (Table S2)), confirming that treatment with tunicamycin for 17 h was effective in inducing ER stress in HepG2 cells. The top downregulated processes in cells exposed to tunicamycin were related to cell cycle and DNA replication, indicating that the treatment arrested cell proliferation. In ER-stressed cells, the regulation of biosynthesis of glycerolipids (e.g., glycerophospholipids and triglycerides) is crucial for maintaining lipid homeostasis by providing phospholipids for cellular membranes and converting excess fatty acids to triglycerides^21^. Therefore, we examined the expression of genes linked to the GO term ‘glycerolipid biosynthetic process’ (GO:0045017) in detail. The results revealed that among the 210 genes implicated in the formation of glycerolipids, *GPAT3* is one of the most strongly activated genes in response to tunicamycin (Fig. 1B, C; Supplementary Information 3 (Table S3)). In contrast to *GPAT3*, the expression of most other genes encoding acyltransferases involved in triglyceride synthesis (e.g., *DGAT2*, *AGPAT2*, *AGPAT3*, *GPAT1*, *DGAT1* and *GPAT4*) was downregulated by tunicamycin (Fig. 1B, C). RT-qPCR (Fig. 2A) and the protein immunoblot (Fig. 2B) confirmed that *GPAT3* expression is upregulated in response to tunicamycin as well as thapsigargin, an alternative inducer of ER stress that inhibits ER Ca^2+^-ATPase.

### ATF4 mediates *GPAT3* upregulation in response to ER stress

In HepG2 cells, ATF4 overexpression has been shown to increase triglyceride content and has been implicated in high fructose-induced lipid deposition^22^. Additionally, ATF4 knockout mice fed a high-fructose or ethanol-containing diet are protected against the development of liver steatosis^23,24^. In previous works, ATF4 deletion in mouse embryonic fibroblasts or HAP1 cells reduced *GPAT3* expression, while ATF4 overexpression in HEK293 cells upregulated *GPAT3*^25–27^. As depicted in Fig. 2C, ER stress inducers tunicamycin and thapsigargin significantly raised the ATF4 protein level in HepG2 cells. To answer the question whether ATF4 plays a role in *GPAT3* upregulation during ER stress in HepG2 cells, we used CRISPR-Cas9 to disrupt the *ATF4* gene. Cells were transfected with gRNAs that direct Cas9 nuclease to cleave either the *ATF4* coding region or, as a control, the hypoxanthine phosphoribosyltransferase (*HPRT*) gene, and exposed to tunicamycin or thapsigargin. *ATF4* mRNA and protein levels were significantly reduced by *ATF4-*targeting gRNAs, compared to cells transfected with gRNA targeting *HPRT* (Fig. 2D, E). Importantly, *ATF4* disruption also decreased *GPAT3* mRNA and protein levels in cells suffering from ER stress (Fig. 2F, G). Thus, the results suggest that ATF4 is involved in transcriptional activation of the *GPAT3* gene in response to ER stress.

### Identification of AP-1 sites as key regulatory elements for the induction of *GPAT3* by ER stress and ATF4

**Fig. 3:**
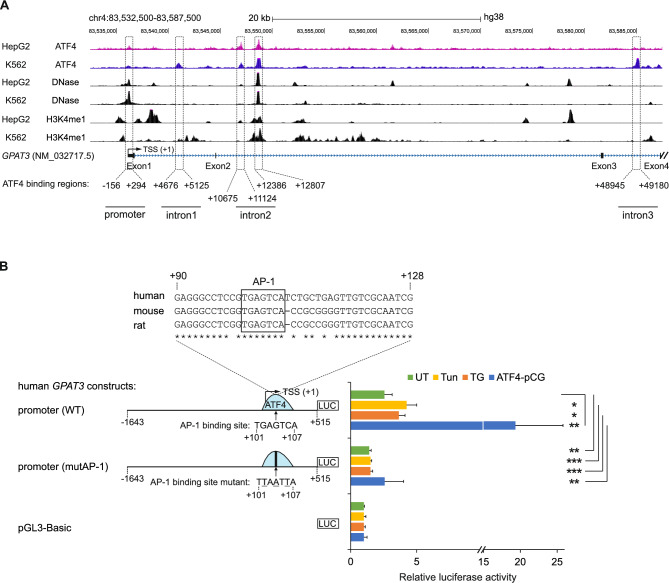
An AP-1 element is involved in the transcriptional activation of the *GPAT3* promoter by ER stress and ATF4 overexpression. (**A**) Genomic coverage tracks visualizing ATF4 ChIP-Seq (ENCODE accessions ENCFF975EWF for HepG2 and ENCFF308UOK for K562), DNase I hypersensitivity (ENCFF113VII for HepG2 and ENCFF972GVB for K562) and H3K4me1 (ENCFF554XSR for HepG2 and ENCFF457URZ for K562) in the region extending from the *GPAT3* gene upstream flank to the beginning of intron 4 (hg38 chr4:83532500-83587500). The *GPAT3* nucleotides are numbered based on the transcription start site (TSS) of the *GPAT3* mRNA NM_032717.5 located at position 83536108. Location of ATF4 binding regions in the *GPAT3* promoter and in the first, second and third intron are shown. (**B**) ER stress and ATF4 upregulate the *GPAT3* gene promoter reporter construct via an AP-1 element. Schematic representation of luciferase constructs and the approximate location of ATF4 ChIP-seq peak (light blue half-ovals). The partial nucleotide sequence of ATF4 ChIP-seq peak in *GPAT3* promoter region along with corresponding mouse and rat genomic sequences aligned by ClustalW is shown. Asterisks below the nucleotide alignment mark sequence identity across species, and the conserved AP-1 element is boxed. HepG2 cells were transfected with luciferase reporter plasmid pGL3-Basic containing either the wild-type *GPAT3* promoter region from −1643 to +515 (WT) or the same region containing inactivating mutations within the AP-1 site (mutAP-1) as shown on the figure. Reporter activities of untreated (UT), tunicamycin (Tun)- or thapsigargin (TG)-treated and ATF4 expression plasmid-transfected (ATF4-pCG) samples are shown as means ± SD from 5 independent transfections, presented relative to the activity of the promoterless reporter plasmid (pGL3-Basic) in the same conditions. * *p* < 0.05, ** *p* < 0.005, *** *p* < 0.0005 comparing the indicated conditions using two-tailed *t* tests followed by Holm-Bonferroni correction.

Publicly available chromatin immunoprecipitation sequencing (ChIP-seq) data shows that ATF4 interacts with several regions of the human *GPAT3* gene, including positions −156 to +294 in the *GPAT3* promoter (nucleotide positions numbered relative to the transcription start site (TSS) of the *GPAT3* RefSeq transcript NM_032717.5, position 83536108 of chromosome 4 of the GRCh38 human reference genome) (Fig. 3A). To study the transcriptional activity of the *GPAT3* promoter, the human genomic DNA fragment spanning positions −1643 to +515, which encompasses the promoter ATF4 binding region, was inserted into the promoterless luciferase reporter plasmid pGL3-Basic and transfected into HepG2 cells. The promoter fragment −1643 to +515 yielded an approximately 2.6-fold elevation in the reporter activity compared to empty pGL3-Basic in unstressed conditions, and a further 1.7-, 1.5- and 7.5-fold increase occurred when the cells were exposed to tunicamycin or thapsigargin or co-transfected with ATF4 overexpression plasmid (ATF4-pCG), respectively (Fig. 3B). The analysis of the *GPAT3* promoter primary structure revealed the presence of the nucleotide sequence 5’-TGAGTCA-3’ at positions +101 to +107, which conforms to the consensus sequence for AP-1 DNA binding motif 5’-TGA(G/C)TCA-3’^28^ and is conserved in mouse and rat (Fig. 3B). To assess the importance of the AP-1 site in the regulation of *GPAT3* expression, inactivating nucleotide substitutions were introduced into the AP-1 element in the promoter fragment −1643 to +515, and reporter assays were performed. The disruption of the AP-1 binding site led to a large reduction in basal, ATF4 overexpression-dependent and ER stress-induced activities of the reporter construct (Fig. 3B).

**Fig. 4:**
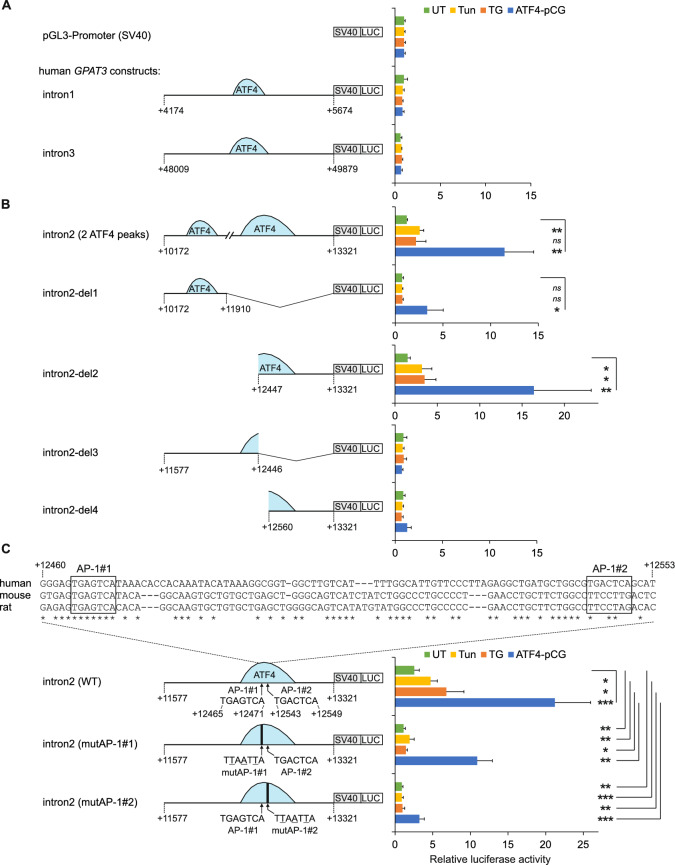
AP-1 elements in the second intron of the* GPAT3* gene are involved in the upregulation of reporter gene expression in response to ER stress and ATF4 overexpression. (**A**, **B**, **C**) HepG2 cells were transfected with luciferase reporter constructs containing candidate regulatory regions from human *GPAT3* introns 1, 2 and 3. Candidate regions were cloned upstream of an SV40 promoter, as depicted on the figure. The nucleotide positions are relative to the *GPAT3* transcriptional start site (numbering as in Fig. 3A), and the approximate locations of ATF4 ChIP-seq peaks are displayed (light blue half-ovals). (**A**) Candidate regions from introns 1 and 3. (**B**) Deletion analysis of the intron 2 candidate region. (**C**) Mutagenesis analysis of the intron 2 candidate region. ClustalW nucleotide sequence alignment between human, mouse and rat for the *GPAT3* intron 2 region containing AP-1#1 and AP-1#2 elements is demonstrated. Asterisks below the alignment indicate sequence identity across species. In the plasmid schematic, nucleotide substitutions engineered to inactivate the AP-1#1 and AP-1#2 sites in the plasmids GPAT3-intron2 (mutAP-1#1) and GPAT3-intron2 (mutAP-1#2) are shown. For all panels, reporter activities of untreated (UT), tunicamycin (Tun)- or thapsigargin (TG)-treated and ATF4 expression plasmid-transfected (ATF4-pCG) samples are shown as means ± SD from 3-5 independent transfections, presented relative to the activity of the pGL3-Promoter plasmid in the same conditions. * *p* < 0.05, ** *p* < 0.005, *** *p* < 0.0005, *ns* (not significant) comparing the indicated conditions using two-tailed *t* tests followed by Holm-Bonferroni correction.

In addition to the promoter, ATF4 ChIP-seq peaks have been detected in introns 1 (peak region from +4676 to +5125 relative to the TSS), 2 (from +10675 to +11124 and +12386 to +12807) and 3 (from +48945 to +49180) of the *GPAT3* gene (Fig. 3A). In order to examine the role of these ATF4 binding sites in the regulation of *GPAT3* expression, a series of fragments of *GPAT3* introns were inserted into luciferase reporter plasmid pGL3-Promoter upstream of an SV40 promoter and transfected into HepG2 cells. Neither the *GPAT3* intron 1 fragment +4174 to +5674 nor the intron 3 fragment +48009 to +49879 (both containing ATF4 binding sites identified by ChIP-seq in K562 cells, but not in the HepG2 cells studied here; Figure 3A) demonstrated the ability to activate the reporter gene compared to the parental vector in unstressed or stressed cells or in response to ATF4 overexpression (Fig. 4A). Differently from that, the fragment +10172 to +13321 encompassing two ATF4 binding regions in intron 2, yielded an approximately 2.5-fold increase in luciferase activity in the presence of tunicamycin and an 11-fold increase in cells transfected with plasmid ATF4-pCG (Fig. 4B). To determine the regulatory element(s) which mediated the ER stress response, a series of *GPAT3* intron 2 deletion constructs were studied. The results revealed that the construct containing the fragment +10172 to +11910 (intron2-del1) is unable to upregulate transcription by ER stress inducers, though it is responsive to ATF4 co-transfection (Fig. 4B). Further deletion analysis using fragments +12447 to +13321 (construct intron2-del2), +11577 to +12446 (intron2-del3) and +12560 to +13321 (intron2-del4) indicated that the transcriptional regulatory element(s) which are activated by ER stress and ATF4 reside in *GPAT3* intron 2 at positions from +12447 to +12560. In terms of chromatin structure, this area overlaps regions of DNase I hypersensitivity and histone 3 lysine 4 monomethylation (Fig. 3A), a marker of enhancers. The inspection of DNA primary structure of this region for possible ATF4-responsive elements revealed the presence of AP-1 site consensus sequences at positions +12465 to +12471 (which we term AP-1#1) and +12543 to +12549 (AP-1#2), the former of which is conserved in mouse and rat (Fig. 4C). To examine the ability of these AP-1 elements to activate transcription, reporter constructs containing the wild-type *GPAT3* intron 2 fragment +11577 to +13321, encompassing the ATF4 binding region with two AP-1 sites (intron2 (WT)), or inactivating mutations of either AP-1#1 (intron2 (mutAP-1#1)) or AP-1#2 motifs (intron2 (mutAP-1#2)), were made and the reporter assays performed. As shown in Fig. 4C, construct intron2 (WT), compared to plasmid pGL3-Promoter, upregulated the reporter activity approximately 2.6-fold in unstressed conditions, and a further 1.9-, 2.7- and 8.3-fold increase occurred in response to tunicamycin, thapsigargin or ATF4 overexpression, respectively. The disruption of AP-1#1 by inactivating nucleotide substitutions resulted in a loss of the basal activity and an approximately 60%, 80% and 50% reduction of inducibility by tunicamycin, thapsigargin or ATF4 overexpression, respectively. At the same time, the disruption of AP-1#2 element totally abolished the transcriptional activation activity of fragment +11577 to +13321 in unstressed and ER stressed cells and only a weak luciferase reporter activity was observed in cells transfected with ATF4-pCG (Fig. 4C). Taken together, the results indicate that the promoter and second intron of *GPAT3* contain AP-1 elements that are transcriptionally responsive to ER stress and ATF4 overexpression.

**Fig. 5:**
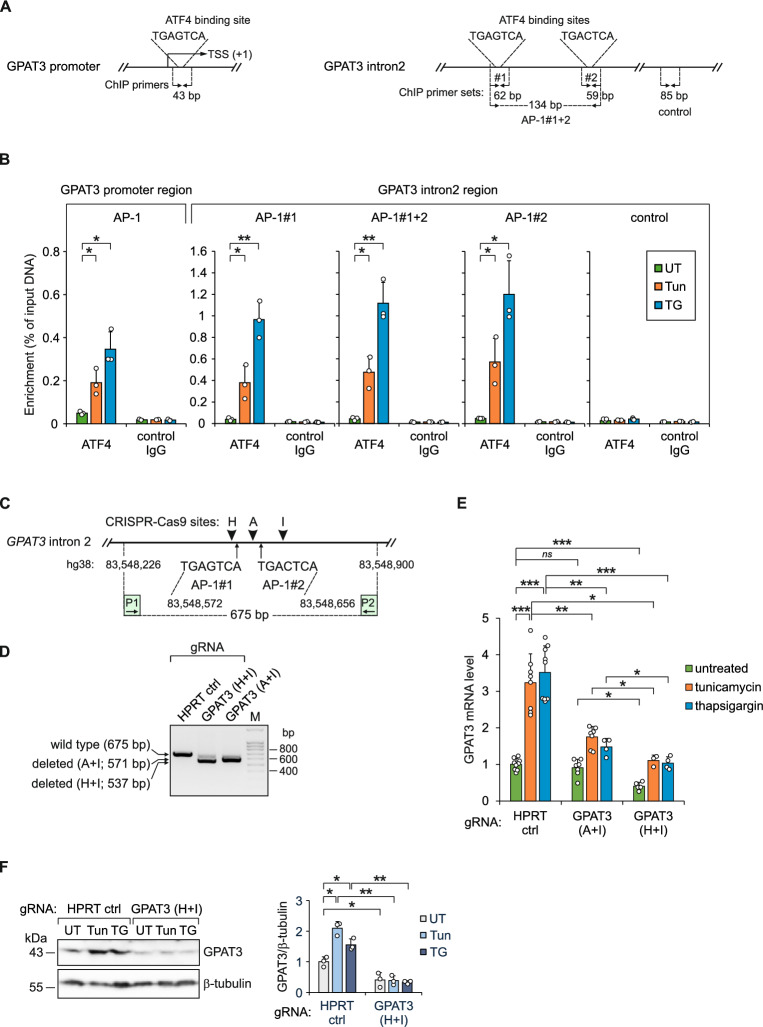
ATF4 binding to *GPAT3* promoter and intron 2 region containing AP-1 sites is increased in native chromatin in response ER stress and deletion of the region from intron 2 downregulates *GPAT3* expression in HepG2 cells. (**A**) Schematic representation of the qPCR amplicons used for ChIP analysis of ATF4 binding to *GPAT3* promoter region containing AP-1 element and *GPAT3* intron 2 region containing AP-1#1 and AP-1#2 elements. The negative control amplicon (designated “control”) is also shown and the sizes of ChIP PCR products are indicated. (**B**) ChIP-qPCR analysis of ATF4 binding to *GPAT3* promoter region containing AP-1 element and *GPAT3* intron 2 region containing AP-1#1 and AP-1#2 elements in cells treated with tunicamycin or thapsigargin for 9 h or left untreated (*n* = 3). An anti-GFP antibody (control IgG) was used as a negative control for immunoprecipitation (*n* = 2). The quantity of immunoprecipitated DNA is presented as percent of input material. The results are shown as the means ± SD. (**C**) Schematic representation of CRISPR-Cas9-based deletion of *GPAT3* intron 2 region containing AP-1#1 and AP-1#2 sites. The location of gRNA target sites (H, A and I) are indicated by filled arrowheads and primers (P1, P2) used for PCR-based analysis of the deletions made in the region by CRISPR-Cas9 are shown. (**D**) PCR-based validation of CRISPR-Cas9 mediated deletions in the *GPAT3* intron 2. Agarose gel of PCR products from the cells transfected with control gRNA (HPRT ctrl) or with *GPAT3* intron 2-specific gRNAs (A+I and H+I). (**E**) RT-qPCR quantification of *GPAT3* mRNA expression in cells transfected with control gRNA (HPRT ctrl) or *GPAT3* intron 2-specific gRNAs (A+I) and (H+I) and treated with tunicamycin or thapsigargin for 17 h or left untreated (*n* = 3-9). Data are presented as the means ± SD and expressed relative to the level of *GPAT3* mRNA in untreated cells transfected with control gRNA. (**F**) The representative immunoblot analysis (*n* = 3) and densitometry quantification of GPAT3 protein expression in HepG2 cells transfected with control gRNA (HPRT ctrl) or *GPAT3* intron 2-specific gRNAs (H+I) and treated with tunicamycin (Tun) or thapsigargin (TG) for 17 h or left untreated (UT). Data are presented as the means ± SD and expressed relative to the level of GPAT3 in untreated cells transfected with control gRNA. * *p* < 0.05, ** *p* < 0.005, *** *p* < 0.0005, *ns* (not significant) comparing the indicated conditions using two-tailed *t* tests followed by Holm-Bonferroni correction. Uncropped gel and blots are presented in Supplementary Information 4.

To explore whether the binding of ATF4 to the *GPAT3* promoter and intron 2 regions containing AP-1 elements is increased in native chromatin of cells suffering from ER stress, we carried out ChIP-qPCR experiments with either an anti-ATF4 antibody or a negative control antibody (anti-GFP), using primer pairs which amplify either the region containing AP-1 site in promoter, the AP-1#1 and AP-1#2 sites in intron 2 or a negative control genomic region in intron 2 outside of ATF4 binding regions according to ChIP-seq (Fig. 5A). As shown in Fig. 5B, a strong enrichment of the PCR amplicons encompassing AP-1 site in promoter and AP-1#1 and AP-1#2 sites in intron 2 was detected in response to the treatment of cells with tunicamycin or thapsigargin in samples immunoprecipitated with anti-ATF4 antibody, while no enrichment was observed in the case of the negative control genomic region or the negative control antibody. Thus, the results indicate that ER stress triggers the binding of ATF4 to the *GPAT3* promoter and intron 2 regions containing AP-1 elements.

### Deletion of the region containing AP-1 sites from the second intron of *GPAT3* gene results in the reduction of *GPAT3* expression, affects the expression of genes involved in inflammation and lipid metabolism and reduces triglyceride level in HepG2 cells

To confirm that the AP-1 elements in the second intron of *GPAT3* participate in the regulation of the endogenous *GPAT3* gene, genome editing of HepG2 cells with CRISPR-Cas9 technology was carried out. Using gRNAs targeting the putative enhancer region, deletions of 104 bp (gRNAs A and I) or 138 bp (gRNAs H and I), encompassing either the AP-1#2 site alone or both AP-1#1 and AP-1#2 sites, respectively, were generated (Fig. 5C, D). Cells transfected with gRNA targeting the *HPRT* gene were used as the control. Following genome editing, cells were treated with tunicamycin or thapsigargin, or left untreated. Consistent with the results of the luciferase reporter assays (Fig. 4C), RT-qPCR revealed that deletion of 104-bp region containing the AP-1#2 element significantly decreases *GPAT3* mRNA expression in stressed cells (Fig. 5E). Moreover, the loss of both AP-1#1 and AP-1#2 sites caused an even larger downregulation of *GPAT3* mRNA in cells under ER stress and reduced *GPAT3* mRNA level also in unstressed cells (Fig. 5E). Thus, the results indicate that both AP-1 elements in intron 2 contribute to the regulation of *GPAT3* gene expression. In agreement with the RT-qPCR data, protein immunoblotting demonstrates that GPAT3 protein level is decreased by the deletion of AP-1#1 and AP-1#2 sites (Fig. 5F).

**Fig. 6:**
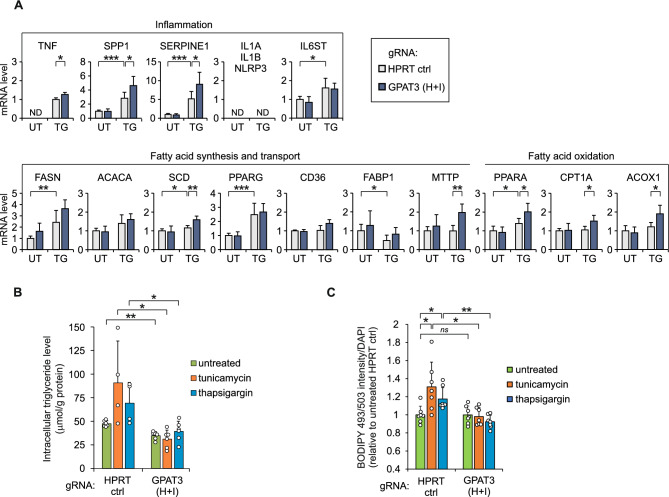
Deletion of the region containing AP-1 sites from the second intron of *GPAT3* affects the expression of genes involved in inflammation and lipid metabolism and reduces triglyceride level in HepG2 cells. (**A**, **B**, **C**) The cells were transfected with gRNAs H and I to delete a 138-bp DNA segment encompassing AP-1#1 and AP-1#2 elements from *GPAT3* intron 2 or with control *HPRT* gRNA. (**A**) RT-qPCR quantification of mRNA levels of selected genes involved in inflammation or lipid metabolism in cells treated with thapsigargin (TG) for 17 h or left untreated (UT) (*n* = 4-9). Data are presented as the means ± SD and expressed relative to the level of *GPAT3* mRNA in untreated cells transfected with control gRNA (for *TNF*, relative to TG-treated control gRNA cells, since the expression was too low to measure in UT cells). (**B**, **C**) Measurement of intracellular triglyceride level (**B**; *n* = 4-6) and fluorometric quantification of neutral lipid content using BODIPY 493/503 staining (**C**; *n* = 7) in cells treated with tunicamycin or thapsigargin for 17 h or left untreated. Triglyceride concentration was normalized to protein concentration (**B**) and fluorescence intensity of BODIPY 493/503 was normalized to nuclear-specific DAPI fluorescence (**C**) in the same sample and data are presented as the means ± SD. * *p* < 0.05, ** *p* < 0.005, *** *p* < 0.0005, *ns* (not significant) using two-tailed *t* tests followed by Holm-Bonferroni correction. In (**A**, **B**, **C**) cells transfected with *HPRT* control gRNA were compared to cells transfected with *GPAT3* gRNA H and I incubated in the same conditions. In addition, in (**A**, **C**) the stress-inducibility was determined comparing treated and untreated *HPRT* Control gRNA cells. ND, not detected.

GPAT3 has been implicated in inflammation and blocking *GPAT3* by siRNA in lipopolysaccharide-stimulated Kupffer cells is accompanied by a reduction of expression of several inflammatory markers, including *TNF*, *IL1A*, *IL1B* and *NLRP3*^12^ . We studied whether the decrease of *GPAT3* expression caused by the deletion of the region containing AP-1 sites from the second intron affects the mRNA level of a set of inflammation-related genes in HepG2 cells. The RT-qPCR data indicate that the treatment of cells with thapsigargin increases the expression of *TNF*, *IL6ST*, *SPP1* and *SERPINE1*, and except *IL6ST*, the expression of the genes is increased more in the cells which were edited by CRISPR-Cas9 to delete the region containing AP-1#1 and AP-1#2 sites from the second intron of *GPAT3* (Fig. 6A). In untreated cells transfected with *GPAT3* gRNAs H and I, the mRNA level of *IL6ST*, *SPP1* and *SERPINE1* is similar to that in the cells transfected with the control *HPRT* gRNA, and *TNF* mRNA is undetectable. The expression of inflammatory markers *IL1A*, *IL1B* and *NLRP3* is below the detection limit both in untreated and thapsigargin-treated HepG2 cells.

We also examined the influence of reduced *GPAT3* expression caused by the deletion of the enhancer from the second intron on the expression of selected genes involved in lipid metabolism. The RT-qPCR analysis reveals that treatment of HepG2 cells with thapsigargin results in the increased expression of several genes participating in fatty acid synthesis, transport and oxidation, such as *FASN*, *SCD*, *PPARG* and *PPARA* (Fig. 6A). Among these genes, the mRNA level of *PPARA*, the major regulator of fatty acid catabolism^29^, was further increased when the *GPAT3* expression is reduced, and a similar change was observed in the case of *SCD*, which encodes an enzyme converting saturated fatty acids into monounsaturated fatty acids^30^. Among the genes unaffected by thapsigargin treatment, *GPAT3* downregulation activated the expression of *MTTP*, which is essential for lipid transfer^31^, and two *PPARA* target genes, *ACOX1* and *CPT1A*, which play key role in fatty acid β-oxidation pathway, encoding the rate-limiting enzyme in the peroxisomal and mitochondrial pathway, respectively^32,33^. In the group of the genes we studied, there were also a couple of genes (*ACACA*, *CD36*) which were unaffected by thapsigargin treatment or reduced *GPAT3* expression. In conclusion, the results suggest that the downregulation of *GPAT3* by the deletion of the enhancer from the second intron may increase fatty acid β-oxidation and may affect certain aspects of fatty acid synthesis and transport.

Previously, an essential role for GPAT3 in triglyceride synthesis has been described^2,17,18^ and, therefore, we studied the impact of *GPAT3* downregulation on the cellular triglyceride level. The genome of HepG2 cells was edited by gRNAs H and I to delete a 138-bp DNA segment encompassing AP-1#1 and AP-1#2 elements, and cells were exposed to ER stress inducer tunicamycin or thapsigargin, or left untreated. As depicted in Fig. 6B, the deletion of the region containing AP-1 elements reduced the triglyceride level in unstressed cells and under ER stress. BODIPY 493/503 staining also demonstrates that in conditions of tunicamycin or thapsigargin exposure, lipid accumulation is lower in the cells transfected with gRNAs H and I, compared to the cells transfected with control gRNA (Fig. 6C). Thus, the data suggests that *GPAT3* upregulation during ER stress potentiates the accumulation of triglycerides within the cell.

## Discussion

The present article reports that *GPAT3* expression is enhanced in response to ER stress and describes the regulatory mechanism underlying the activation of the gene. We demonstrate that the exposure of cells to ER stress inducers increases ATF4 protein level and ATF4 binding to chromatin, which triggers the upregulation of *GPAT3* transcription via AP-1 elements in the promoter and in the second intron of the gene. The AP-1 transcription factors function as pairwise combinations JUN, FOS, MAF and ATF protein family members^34^. These proteins play a crucial role in many cellular processes, including proliferation, differentiation and response to stress^34–36^, and our findings are in line with the studies showing that AP-1 transcription factors contribute to the regulation of gene expression by operating in promoters as well as in distal enhancer regions^37,38^. The AP-1 dimeric complexes are formed through a leucine zipper motif, and several AP-1 components have been identified as ATF4 dimerization partners^39,40^. In line with this, ChIP-seq has revealed chromatin binding profile similarity between ATF4 and AP-1 family members JUN and JUND in HepG2 cells^25^. However, the prevalent ATF4 DNA binding sequence motif in cells under ER stress appears to be the C/EBP-ATF composite site, which is present in approximately 70% of ATF4 ChIP-seq peaks, while the AP-1 motif is found in a significantly smaller fraction, approximately 15%, of ATF4 peaks^25^. Many publications have characterized gene expression regulation by ATF4 via C/EBP-ATF composite sites during ER stress, such as detailed studies for genes including *TRIB3*, *DDIT3*, *ASNS*^41–43^, but previous knowledge about the ATF4-mediated regulation via AP-1 sites is scarce.

The analysis of transcriptome revealed that the inducibility of *GPAT3* in response to tunicamycin in HepG2 cells is unique among the genes encoding the members of the GPAT family. A similar pattern has previously been observed in the case of *GPAT3* induction in mouse liver by corticosterone, a ligand for glucocorticoid receptor (GR), which acts via GR elements in *GPAT3* promoter^17^. While corticosterone treatment significantly increased the mRNA and protein expression of GPAT3, the mRNA expression of other GPAT isoforms was not changed^17^. Also, *GPAT3* mRNA level rose up to 7-fold during calcium-stimulated human keratinocyte differentiation, but, in contrast, *GPAT1* and *GPAT4* mRNA levels decreased approximately 50%^44^. In addition, *GPAT3*, but not *GPAT4*, is upregulated in fatty livers of ob/ob mice by the administration of PPARγ agonist rosiglitazone^2,45^. Thus, GPAT3 is the major inducible GPAT isoform in several situations where the expression level of the other members of the GPAT family is reduced or remains unchanged. Given our finding that *GPAT3* expression is positively regulated by ATF4, in future works it could be worthwhile to evaluate *GPAT3* expression levels under further types of stressors, as ATF4 is the master regulator of the ISR induced by a variety of stresses^46^, including oxidative stress, nutrient deprivation, double-stranded RNA and heme deficiency, in addition to the endoplasmic reticulum stress conditions studied here. The regulatory interaction of ATF4 with the *GPAT3* gene could be more complex in some of these conditions, as ATF4 ChIP-Seq revealed multiple ATF4 binding sites, including some which were cell type-specific.

Current study is performed in hepatocellular cancer cell line, and MASLD and hepatocellular cancer are closely related^47,48^, as MASH, the severe form of MASLD, has the potential to progress to hepatocellular cancer. During the development of hepatocellular cancer from MASLD, changes occur in cell metabolism and in the expression of many genes, including genes involved in lipid metabolism^49,50^. Therefore, it is necessary to mention that further studies with primary hepatocytes are needed in order to confirm that the findings described in this article take place also in MASLD.

Taken together, data presented in this paper demonstrate that in cells suffering from ER stress, as well as in unstressed cells, the downregulation of *GPAT3* expression by the deletion of an ATF4-binding enhancer element in the second intron results in a decrease of triglyceride level, clarifying the regulatory mechanism behind previous reports describing the pivotal role of GPAT3 in triglyceride synthesis.

## Materials and methods

### Mammalian cell culture and treatment

HepG2 cells (obtained from the American Type Culture Collection) were cultured in Dulbecco’s Modified Eagle Medium (4.5 g/l D-glucose; Gibco) supplemented with 10% fetal bovine serum and 1× penicillin-streptomycin. To induce ER stress, the cells were exposed to 2.5 µg/ml tunicamycin (with the exception of 2 µg/ml tunicamycin used in RNA-seq experiment) or 0.5 µM thapsigargin for 17 h (with the exception of 9 h used in ChIP analysis). Both chemicals were purchased from Sigma.

### RNA-seq and data analysis

RNeasy Midi Kit in combination with the on-column DNase digestion (Qiagen) was used to extract total RNA from HepG2 cells untreated or treated with 2 μg/ml tunicamycin for 17 h^25^. RNA-seq libraries were prepared from 250 ng of total RNA using the Lexogen QuantSeq 3′ mRNA-Seq Library Prep Kit for Illumina (FWD) as described in^51^. Libraries were amplified for 14 cycles to add the Illumina adapters and i7 index (7015-7024, Lexogen) and sequenced at the Estonian Genome Center Core Facility.

Sequencing reads were processed using the nf-core RNA-Seq pipeline (version 1.4.2)^52^ with the hg19 reference genome and Ensembl release 87 transcript definitions with the default settings. The filterByExpr function from the edgeR package (version 3.24.3)^53^ was used to remove lowly expressed genes, requiring at least 10 total counts and at least 2 counts in some samples, resulting in 12733 genes remaining. Differential gene expression analysis was carried out using DESeq2 (version 1.30.1)^54^ with default parameters. *P* values were adjusted for multiple testing using the Benjamini-Hochberg false discovery rate method and *P*_adj_ < 0.05 was considered significant. GO biological process enrichment analysis was carried out using the g:Profiler webtool (date accessed: May 13, 2025)^55^ to query the top 500 up- and downregulated genes (ranked based on statistical significance). The ’driver terms’ analysis implemented in g:Profiler was used select terms to plot (reducing similar GO terms). Raw sequencing data for RNA-Seq has been deposited to NCBI GEO (accession: GSE296996).

### Plasmids

To generate the reporter constructs for the analysis of transcriptional regulation of human *GPAT3*, fragments from the 5’-end and intronic regions of the *GPAT3* gene were amplified by PCR from DNA isolated from HepG2 cells and inserted into the firefly luciferase plasmid without promoter (pGL3-Basic) and with SV40 promoter (pGL3-Promoter) (both from Promega), respectively. The different fragments studied extended from position -1643 to +515 (the construct is named promoter (WT)), +4174 to +5674 (intron1), +10172 to +13321 (intron2 (2 ATF4 peaks)), +10172 to +11910 (intron2-del1), +12447 to +13321 (intron2-del2), +11577 to +12446 (intron2-del3), +12560 to +13321 (intron2-del4), +11577 to +13321 (intron2 (WT)) and +48009 to +49879 (intron3) (*GPAT3* nucleotides are numbered based on the transcription start site (TSS) of the *GPAT3* mRNA NM_032717.5 located at position 83536108 of chromosome 4 of the GRCh38 human reference genome). Oligonucleotide-directed mutagenesis was used to inactivate potential regulatory elements identified during the analysis. In order to mutate the putative AP-1 site located at positions +101 to +107 in the construct promoter (WT) and +12465 to +12471 in the construct intron2 (WT), the nucleotide sequence 5’-TGAGTCA-3’ was replaced with 5’-TTAATTA-3’ (the substituted nucleotides are underlined), creating constructs promoter (mutAP-1) and intron2 (mutAP-1#1). To mutate the putative AP-1 site located at +12543 to +12549 in the construct intron2 (WT), the nucleotide sequence 5’-TGACTCA-3’ was replaced with 5’-TTAATTA-3’, creating construct intron2 (mutAP-1#2). The plasmids were verified by Sanger sequencing.

### Dual-luciferase reporter assay

Cells grown in 96-well plates were cotransfected with firefly luciferase plasmid (either pGL3-Basic, pGL3-Promoter or constructs derived from these plasmids by the insertion of *GPAT3* gene fragments) and *Renilla* luciferase plasmid (pRL-TK; Promega) using polyethylenimine (PEI-MAX 40,000; Polysciences Inc. #24765) as described previously^56^. Where indicated, expression plasmid encoding human ATF4 (ATF4-pCG)^57^ or the corresponding empty vector (pCG) was included in the transfection mixture. The cells were treated with ER stress inducer (tunicamycin or thapsigargin) 18 h after transfection or left untreated. All conditions were applied to the cells in duplicate wells and repeated in at least three independent experiments.

Firefly and *Renilla* luciferase activities were measured by a Promega dual-luciferase reporter assay as described previously^41^. Firefly luciferase activity was normalized to the *Renilla* luciferase activity in each sample, and the normalized luciferase activities are presented relative to the results from the empty vector (either pGL3-Basic or pGL3-Promoter).

### CRISPR-Cas9-mediated genome editing in cell culture

Alt-R CRISPR-Cas9 System (crRNA-tracrRNA duplexes and S. p. Cas9 nuclease; Integrated DNA Technologies (IDT)) was used to generate mutations in *ATF4* or *GPAT3* genes. The solutions of crRNA-tracrRNA duplexes (gRNAs) and ribonucleoprotein (RNP) complexes were made as described previously^25^.

In order to disrupt the *ATF4* gene, the cells were transfected with the equimolar mixture of gRNA B (containing sequence 5’-AGAUGACCUUCUGACCACGU-3’) and C (5’-AGGUCUCUUAGAUGAUUACC-3’). To delete from the genome the enhancer region of *GPAT3* intron 2 containing both AP-1#1 and AP-1#2 sites or the region containing only AP-1#2 site, the cells were transfected with the equimolar mixture of gRNAs consisting of gRNA I (5’-GCAUGGGGUAAGACUUGCAG-3’) and either gRNA H (5’-GGUGUUUAUGACUCACUCCC-3’) or gRNA A (5’-CCACAAAUACAUAAAGGCGG-3’), respectively. A predesigned Alt-R CRISPR-Cas9 crRNA (IDT) targeting the human *HPRT* gene was used in control experiments.

The RNP complexes were introduced by electroporation into 2×10^5^ HepG2 cells resuspended in Buffer R, using 10 µl tips with one pulse at a setting 1350 V / 30 ms (Neon Transfection System, Invitrogen). Transfected cells were cultured in antibiotic-free medium for 24 h and then treated with tunicamycin or thapsigargin or left untreated for a further 17 h. Biological replicates were obtained from independently transfected cells cultured in separate wells of the cell culture plate.

To estimate CRISPR/Cas9-mediated deletion efficiency in *GPAT3* intron 2, genomic DNA was extracted and the PCR amplification was performed with Phusion Hot Start II High-Fidelity DNA Polymerase (Thermo Scientific) using the primers 5’-CTCCAGCCATAGGACAAATGCATCTCC-3’ (named P1) and 5’-ACCTGAATTCTCTCCAAATAGGTATAGCAACC-3’ (P2). The PCR products were resolved in ethidium bromide-stained 1.5% agarose gel.

### Chromatin immunoprecipitation

Chromatin immunoprecipitation experiments were performed as described previously^51^, with the following modifications. HepG2 cells were crosslinked with 1% formaldehyde for 10 min at room temperature and then quenched with 0.125 M glycine. Fixed cells were treated with nuclei isolation buffer, resuspended in ChIP lysis buffer and sonicated with Bioruptor Plus (Diagenode) for 15 cycles (30 s on, 60 s off) at 4 °C with power set to high. Chromatin immunoprecipitation was performed using rabbit anti-ATF4 polyclonal antibody (Invitrogen #PA5-27576) or rabbit anti-GFP polyclonal antibody (Clontech #8367-1, Mountain View, CA, USA) as a negative control. Immunocomplexes were isolated by Protein G sepharose beads (Cytiva), chromatin crosslinking was reversed and DNA was purified using QIAquick PCR Purification Kit (Qiagen, Hilden, Germany). Enriched ChIP signals relative to input chromatin were quantified by RT-qPCR using the ChIP-qPCR primers presented in Supplementary Information 5 (Table S4).

### RT-qPCR

Total RNA was extracted from cells using TRIzol (Invitrogen), treated with DNase I and used for cDNA synthesis with FIREScript reverse transcriptase (Solis BioDyne) according to the manufacturer’s instructions. Real-time quantitative PCR was performed as described previously^58^ and glyceraldehyde-3-phosphate dehydrogenase (*GAPDH*) mRNA was used as the endogenous reference for expression level normalization. The primer pairs used are presented in Supplementary Information 5 (Table S4).

### Western blotting

Immunoblotting was carried out as described previously^59^. Total protein was extracted using Laemmli buffer and cell lysates were boiled at 100°C for 5 min. Protein concentration in cell lysates was determined by the Pierce BCA protein assay. The following primary antibodies were used: rabbit anti-GPAT3 polyclonal antibody (1:1000 dilution; Proteintech #30765-1-AP), rabbit anti-ATF4 polyclonal antibody (1:2000 dilution; Invitrogen #PA5-27576) and rabbit anti-β-tubulin polyclonal antibody (1:2500 dilution; Abcam #ab6046). Antibody binding was detected by the horseradish peroxidase-conjugated goat anti-rabbit IgG secondary antibody (1:5000 dilution; Cell Signaling Technology #7074). Blots were treated with Immobilon chemiluminescent reagent (EMD Millipore) and protein signals were visualized and quantified with ChemiDoc XRS+ detection system and Image Lab software (BioRad).

### Intracellular triglyceride quantification

Total triglyceride levels were determined using the triglyceride quantification kit (Sigma #MAK266) following the manufacturer’s instructions. Transfected HepG2 cells were lysed in 5% NP-40 and triglycerides were solubilized by repeated heating at 96°C for 5 min. Fluorescence was measured on a GENios Plus (Tecan, Switzerland) microplate reader (excitation, 540 nm; emission, 595 nm) and the background fluorescence of samples without lipase treatment was subtracted from the sample readings. Protein concentrations were determined using the BCA protein assay (Pierce). The concentration of triglycerides was calculated from the standard curve and was normalized to protein levels.

### Measurement of cellular neutral lipids by fluorometry

Transfected HepG2 cells were washed once with Dulbecco’s phosphate-buffered saline (PBS; Gibco), collected and fixed with 4% paraformaldehyde for 10 min. Cells were washed with PBS and stained with 10 μM BODIPY 493/503 (Molecular Probes) and 0.5 μg/ml DAPI (Boehringer Mannheim) in 300 μl PBS for 30 min at room temperature in the dark. After staining, cells were thoroughly washed and resuspended in PBS. Samples were loaded into a flat-bottom black 96-well plate in technical quadruplicates (50 μl per well) and fluorescence intensity (Ex/Em = 485 nm/520 nm for BODIPY 493/503; Ex/Em = 350 nm/450 nm for DAPI) was measured using a microplate reader Infinite M200 PRO with i-control software (Tecan). In each sample, the quadruplicate measurements were averaged, and the average fluorescence of unstained cells was subtracted, and the fluorescence reads of BODIPY 493/503, a stain for neutral lipids, were normalized to the fluorescence reads of DAPI, a stain for nuclear quantification. The normalized BODIPY 493/503 values are presented relative to the values of control gRNA-transfected untreated cells.

## Supplementary Information

Supplementary Information 1.

Supplementary Information 2.

Supplementary Information 3.

Supplementary Information 4.

Supplementary Information 5.

## Data Availability

Raw sequencing data for RNA-Seq has been deposited to NCBI GEO (accession: GSE296996).
