# Supplementary figures and images for: AP-1 elements in the promoter and second intron mediate endoplasmic reticulum stress-induced expression of the GPAT3 gene
Source: Sci Rep. 2025 Dec 14;16:2730. doi: 10.1038/s41598-025-32503-y (PMC12824169; doi:10.1038/s41598-025-32503-y)

Cropped images of Western blots for Figures 2B (A), 2C (B), 2E (C), 2G (D), 5D (E) and DNA agarose gel for Figure 5B (F).

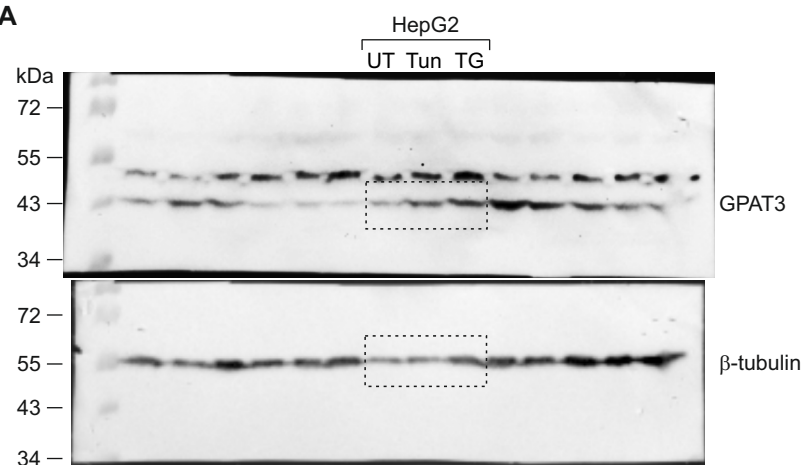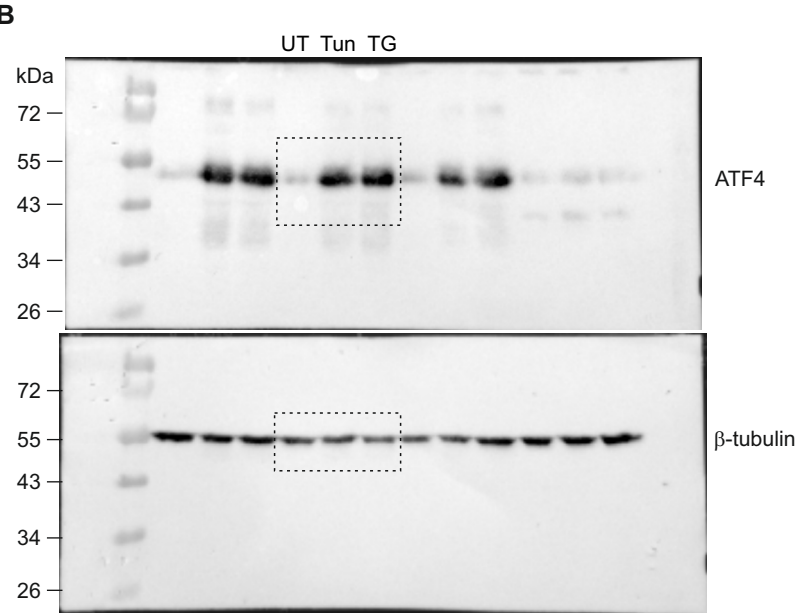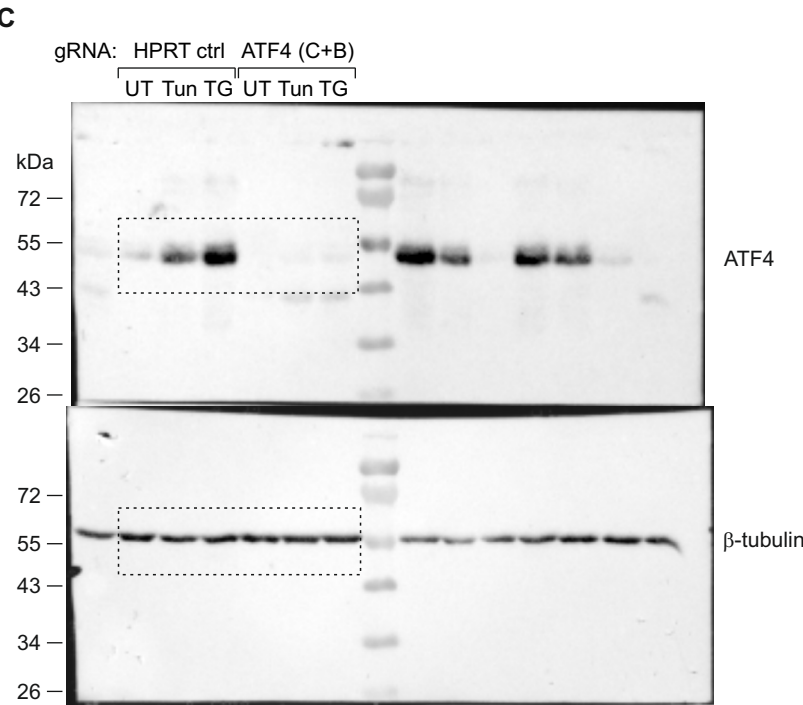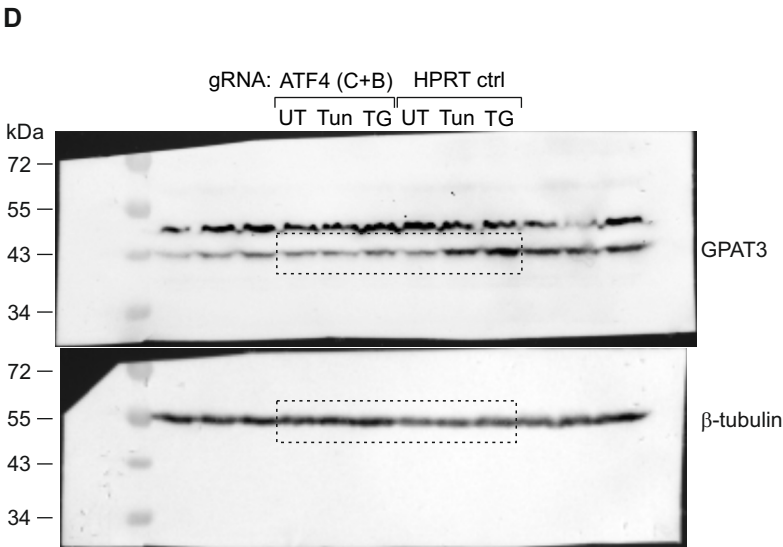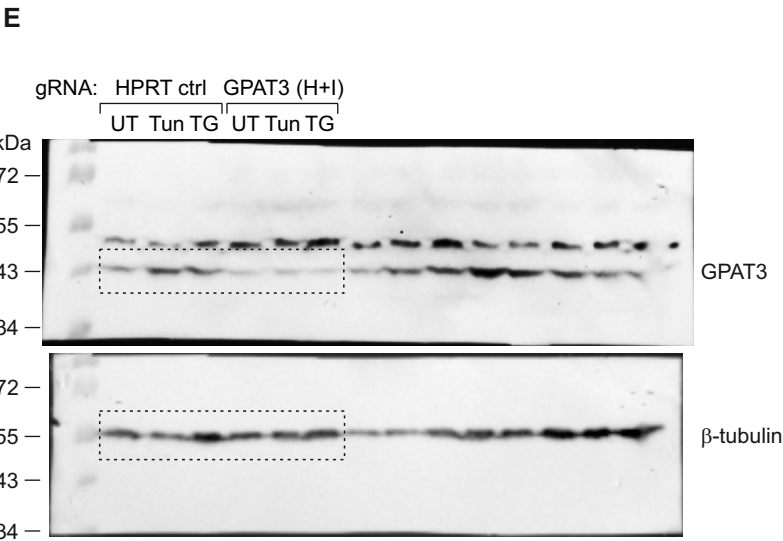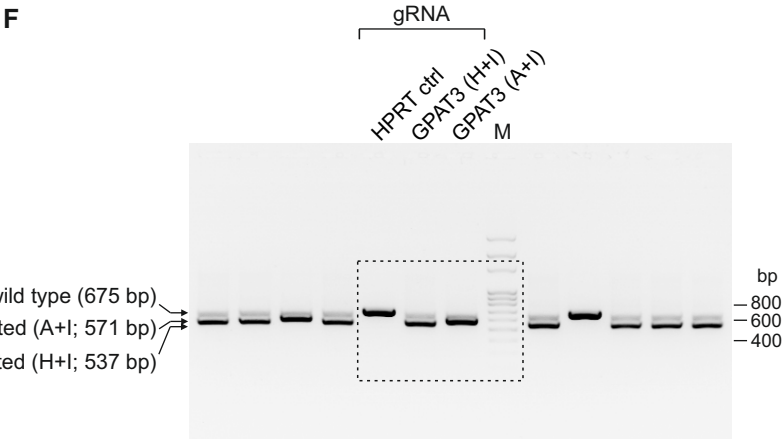

Supplement: Supplementary file 4 — Supplementary Information 4. [file 41598_2025_32503_MOESM4_ESM.pdf]
